# Assessing Vascular Tone and Fluid Balance in Septic and Cardiogenic Shock: A Feasibility Study on Skin Water Loss as a Diagnostic Tool

**DOI:** 10.3390/biomedicines13112644

**Published:** 2025-10-28

**Authors:** Sabrina Kopp, Ingo Sagoschen, Susanne Helena Karbach, Martin Russwurm, Philipp Lurz, Thomas Münzel, Johannes Wild

**Affiliations:** 1 Department of Cardiology-Cardiology I, University Medical Center Mainz, 55131 Mainz, Germany; 2 German Center for Cardiovascular Research (DZHK)–Partner Site RheinMain, 55131 Mainz, Germany; 3 Center for Thrombosis and Hemostasis, University Medical Center Mainz, 55131 Mainz, Germany; 4 Department of Internal Medicine and Nephrology, University Hospital Marburg, 35043 Marburg, Germany; 5 Institute of Pharmacology, University Marburg, 35043 Marburg, Germany

**Keywords:** fluid management, transcutaneous water loss, skin barrier, septic shock, cardiogenic shock

## Abstract

**Background/Objectives**: Fluid management in shock remains a clinical challenge, with ongoing debate about optimal guidance. Despite advanced technologies, fluid balance assessment is often inadequate. The SkInShock study investigated whether transepidermal water loss (TEWL) measurements could improve fluid balance estimation and serve as a non-invasive marker of vascular tone in patients with septic or cardiogenic shock. **Methods**: In this prospective single-center feasibility study (DRKS00027981), TEWL was measured daily in eight mechanically ventilated patients using a Tewameter^®^ (Courage+Khazaka, Cologne, Germany), which quantifies transcutaneous water evaporation. Total daily skin water loss was calculated either via direct TEWL measurements or an estimation formula (6 mL/kg/day + 20%/°C deviation from 37 °C). Systemic vascular resistance index (SVRI) was measured simultaneously using PiCCO^®^ technology (Pulsion Medical Systems, Munich, Germany) to evaluate the relationship between TEWL and vascular tone. **Results**: TEWL values were consistent across most body sites, except the forehead. TEWL-based estimates of skin water loss were significantly lower than formula-based estimates (*p* < 0.01). Formula-based values overestimated water loss at low TEWL levels and underestimated it at higher levels, with deviations reaching ±100%. While absolute TEWL values did not correlate with SVRI, intra-individually normalized values showed a significant negative correlation, indicating that higher skin water loss corresponded to lower vascular tone. **Conclusions**: TEWL measurement is feasible in ICU patients and may enhance fluid balance assessment and vascular tone monitoring. Our preliminary findings indicate that this non-invasive method could complement current diagnostics but warrants further investigation in larger cohorts.

## 1. Introduction

Fluid therapy management in septic and cardiogenic shock remains a topic of debate in intensive care medicine. While optimal intravenous fluid therapy is essential for shock treatment [1,2,3], determining the appropriate fluid volume and balancing resuscitation with fluid removal remains a challenge [4]. In septic shock, large positive fluid balances are often necessary for hemodynamic stabilization. In contrast, in cardiogenic shock, circulatory support with either vasoactive agents or mechanical support is prioritized over fluid therapy [5,6]. Optimizing intravascular volume and detecting fluid overload remain crucial in both conditions. The S3 guideline “Intravascular Volume Therapy in Adults” [7] recommends guiding fluid therapy based on dynamic preload indices, such as stroke volume variation and pulse pressure variability, rather than static parameters like central venous pressure [8]. A standardized passive leg raise maneuver and ultrasound-based assessments are also advised for evaluating volume responsiveness. Overall, clinical evaluation remains the cornerstone of fluid status assessment [7].

In intensive care, fluid balance documentation focuses primarily on administered fluids and urinary output. At the same time, trans-epithelial losses through the lungs and skin are estimated via approximation formulas, leading to potential inaccuracies [9,10]. The skin as the body’s largest organ, plays a vital role in thermoregulation [11] and water homeostasis [12]. Clinical assessments of skin turgor and mucous membranes are commonly used for fluid status estimation [7], yet non-invasive, objective measurements of skin function are not standard in ICU practice. While cutaneous perfusion changes have been explored as a diagnostic tool in septic shock [13,14], the potential of transepidermal water loss (TEWL) monitoring in septic or cardiogenic shock remains unexplored. Beyond diseases that compromise the integrity of the stratum corneum, such as atopic dermatitis or psoriasis (24), processes that promote vasodilation and enhanced cutaneous blood flow also lead to increased TEWL [15]. Accordingly, TEWL is associated with and might serve as an indicator of vasodilation.

In our SkInShock study, we aimed to test the hypothesis that the skin as an easily accessible organ, could be used for an optimized, noninvasive assessment of vascular tone and fluid balance in septic or cardiogenic shock.

## 2. Materials and Methods

### 2.1. Study Design and Patient Selection

This prospective, single-center feasibility study (German Clinical Trials Register ID: DRKS00027981) was approved by the Ethics Committee of the Landesärztekammer Rheinland-Pfalz (Ethics approval number: 2021-16170, issued on 2 December 2021). The study was conducted in the medical intensive care unit of the Centre for Cardiology, University Medical Centre Mainz. Eight mechanically ventilated patients were included, comprising three patients with cardiogenic shock and five with septic shock (see Table 1 for extensive baseline characteristics).

As this was designed as a prospective feasibility study, all intubated patients with septic or cardiogenic shock who were admitted to the ICU and did not meet the exclusion criteria below were included in the study upon admission to the ICU. As all patients in the study were intubated, informed consent was obtained when an informed consent interview was reasonable in the individual case or a legal guardian/proxy had been appointed. As no additional invasive measures were carried out in the context of the study and the instrumental diagnostics used consisted exclusively of measurements with medical devices within the scope of their approval, subsequent inclusion was ruled possible and suggested by the local Ethics Committee. Cutaneous infection focus, burns, pregnant women or any acute skin diseases/lesions were defined as exclusion criteria.

### 2.2. Measurement of Transepithelial Water Loss (TEWL)

Transepithelial water loss (TEWL) was assessed once daily at four anatomical sites: the forehead, upper limbs, trunk, and lower limbs. The positions were selected because they also are used by Wallace’s rule of nines [16], which formed the scientific basis for the subsequent calculations. Measurements were obtained daily from the time of admission until extubation or death, resulting in an individual observation period ranging from 2 to 6 days (All corresponding measurement time points are provided in the Supplemental Table S1). TEWL was measured using the Courage & Khazaka Multi Display Device (MDD 4, Courage+Khazaka, Cologne, Germany) in combination with the Tewameter TM300 (Courage+Khazaka, Cologne, Germany). The Tewameter TM300 uses an open-chamber principle with a hollow cylindrical probe (length 2 cm, diameter 1 cm) including two pairs of sensors which record relative humidity and temperature gradients above the skin surface. These measurements enable indirect calculation of the water vapor density gradient, from which TEWL is derived. The device provides high-resolution recordings with a measurement resolution of ±0.01% for humidity, ±0.01 °C for temperature, and 0.1 g/h/m^2^ for TEWL. Measurements are valid within an operating range of 10–40 °C and 30–70% relative humidity. The specific device has been extensively used and validated in prior research [12,17,18,19]. Data acquisition was performed at one-second intervals, in accordance with the manufacturer’s guidelines, until the standard deviation of the current value relative to the running mean was <0.2. TEWL was subsequently calculated by the Multi Display Device (MDD 4, Courage+Khazaka, Cologne, Germany) based on Fick’s law of diffusion, using the measured humidity and temperature values. Environmental conditions (room temperature and relative humidity) were automatically recorded by the system during each measurement. Each measurement at a single site required approximately 2–4 min, depending on the time needed for the defined standard deviation to reach a stable value <0.2. In our experience, the operator’s experience in handling the device substantially influenced this duration, since an upright positioning of the measuring head and adequate contact pressure are necessary for a stable measurement. The acquisition of measurements at all four body sites therefore took about 30 min, including repositioning between measurement locations. Considering setup, device initialization, cleaning, and dismantling, a total of approximately 45 min was needed for a complete and thorough measurement session.

Total daily water loss through the skin was calculated using direct TEWL measurements or the established estimation formula by Cox et al. [9] as described here: Total transcutaneous water loss = 10 mL/kg body weight/24 h + 20% correction per °C deviation from 37 °C. As all patients were intubated and mechanically ventilated with active humidification, only cutaneous water loss was considered relevant for insensible water loss assessment, and therefore the result was reduced by 40% as suggested by Cox et al. [9].

Direct TEWL-derived estimation: Summation of individual site measurements (the forehead, upper limbs, trunk, and lower limbs) in g/h/m^2^ × 24 h extrapolated to total body surface area. We adapted the Wallace rule of nines for extrapolation to total body surface area [16]. Total transcutaneous water loss = TEWL_measured_(lower limb) × 0.18 × 2 + TEWL_measured_(upper limb) × 0.09 × 2 + TEWL_measured_(head) × 0.09 + TEWL_measured_(trunk) × 0.36.

### 2.3. Hemodynamic Evaluation

Systemic vascular resistance index (SVRI) was measured simultaneously using the PiCCO^®^ technology (Pulsion Medical Systems, Munich, Germany) to investigate the relationship between skin water loss and vascular tone. TEWL and SVRI data were collected concurrently to assess potential correlations between transcutaneous water loss and systemic perfusion parameters. In two patients—one presenting with septic shock and one with cardiogenic shock—the clinical team did not identify an indication for PiCCO^®^ catheter placement. In accordance with the ethics committee’s ruling, additional invasive procedures conducted exclusively for the purposes of this feasibility study were not authorized, and consequently, no measurement data were collected for these patients (All individual measurements are provided in Supplemental Table S1).

### 2.4. Statistical Analysis

Statistical analyses were performed using GraphPad Prism software (version 10; GraphPad Software Inc., San Diego, CA, USA). Data are presented as individual values or mean  ±  standard error of the mean (SEM). Normality was assessed using the Kolmogorov–Smirnov test. Descriptive statistics for relevant patient characteristics are presented as mean with standard deviation (SD) or absolute numbers with corresponding percentages. Additional analyses included 1-way ANOVA with Holm-Šídák’s multiple comparisons test, Wilcoxon test or Welch’s *t*-Test and simple linear regression where appropriate. A *p*-value of <0.05 was considered statistically significant, with significance levels denoted as follows: * *p*  <  0.05; ** *p*  <  0.01; *** *p*  <  0.001.

## 3. Results

We recruited eight critically ill patients for this feasibility study. Five of them presented with septic shock and three with cardiogenic shock. The mean age of the participants was 64.3 ± 9.3 years, and 12.5% were female (see Table 1 for Baseline Characteristics). The average body mass index (BMI) was 26.24 ± 5.11 kg/m^2^. Common comorbidities included chronic kidney disease (62.5%), dyslipoproteinemia (62.5%), hypertension (50%) and chronic heart failure (50%). Initial laboratory parameters showed a mean leukocyte count of 14.84 ± 10.4 × 10^9^/L, platelet count of 220.88 ± 141.8 × 10^9^/L and haemoglobin of 10.57 ± 2.27 g/dL. Mean serum creatinine on ICU admission was 1.83 ± 1.25 mg/dL, total bilirubin 0.90 ± 0.61 mg/dL, aspartate aminotransferase 194.63 ± 152.0 U/L), alanine aminotransferase 93.13 ± 62.2 U/L), and lactate 3.46 ± 2.2 mmol/L (see Supplemental Table S1 for all individual measurements).

During ICU treatment, we measured a lowest mean platelet count of 116.00 ± 83.6 × 10^9^/L (see Table 2 for Data about Clinical course and outcomes). The peak serum creatinine was 3.23 ± 2.5 mg/dL, and the highest total bilirubin was 1.99 ± 2.3 mg/dL. Inflammation or tissue injury was reflected by a peak C-reactive protein of 227.38 ± 60.9 mg/L, creatine kinase at 1574.25 ± 1680.4 U/L, and troponin levels reaching 59,820.88 ± 142,115.2 pg/mL The highest lactate of the examined patients was 6.51 ± 4.2 mmol/L. Severity of illness was reflected by a mean maximum APACHE II score of 27 ± 8 and a maximum SOFA score of 12 ± 3. The average duration of mechanical ventilation was 167.40 ± 160.2 h. Patients remained in the ICU for an average of 245.65 ± 197.1 h, with a total hospital stay of 26.50 ± 15.8 days. Overall, 62.5% of patients were transferred alive from the ICU, and the same proportion was also discharged alive from the hospital.

Twenty-four-hour fluid intake and excretion data were stratified by type of shock, with 14 measurements collected from the five patients with septic shock and 12 from the three patients with cardiogenic shock (Table 3). As expected, patients with septic shock had significantly higher parenteral fluid intake than those with cardiogenic shock (4581 ± 1337 mL vs. 3012 ± 715.1 mL, *p* = 0.001), while enteral intake did not differ significantly between groups. Total fluid intake was also significantly greater in the septic shock group (4718 ± 1412 mL vs. 3365 ± 898.9 mL, *p* = 0.01). Urine output was comparable between groups, but ultrafiltrate volumes were significantly higher in the cardiogenic shock group (1728 ± 1454 mL vs. 425.8 ± 584.7 mL, *p* = 0.03). Skin water loss, whether calculated via formula or measured by TEWL, did not significantly differ between groups. When total fluid output was calculated using formula-based skin water loss, it was significantly higher in cardiogenic shock patients (2646 ± 1144 mL vs. 1721 ± 652.8 mL, *p* = 0.02), a finding mirrored when using TEWL-based estimates (2521 ± 1080 mL vs. 1650 ± 678.6 mL, *p* = 0.02). Consequently, the 24 h fluid balance was significantly more positive in septic shock patients, both using formula-based (2997 ± 1492 mL vs. 719.7 ± 1564 mL, *p* = 0.001) and TEWL-based (3068 ± 1428 mL vs. 844.6 ± 1502 mL, *p* = 0.002) calculations.

**Figure 1:**
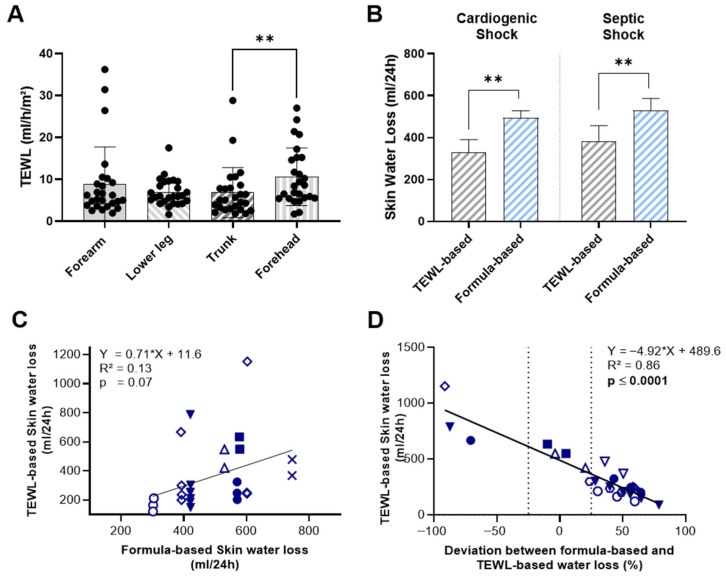
Comparison of TEWL-Based and Formula-Based Estimates of Skin Water Loss in ICU-patients with cardiogenic or septic shock. (**A**) TEWL measurements at the forearm, lower leg, trunk and forehead by measurement site. (**B**) 24 h skin water loss estimated by TEWL measurements and formula-based calculations. (**C**) Correlation between TEWL-based and formula-based estimates of 24 h skin water loss. (**D**) Correlation between TEWL-based 24 h skin water loss estimates and the deviation from formula-based estimates. Identical symbols represent repeated measurements in the same patient; color-filled symbols indicate patients with cardiogenic shock. Data include 12 individual measurements from 3 patients with cardiogenic shock and 14 from 5 patients with septic shock. Statistical analysis was performed using Holm-Šídák’s multiple comparisons test (**A**), Wilcoxon matched-pairs signed-rank test (**B**) and simple linear regression (**C**,**D**). ** *p*  <  0.01.

TEWL measurements at the forearm, lower leg, and trunk did not show significant variation by measurement site, except for skin values at the forehead, which were significantly higher than those recorded at the trunk (10.61 ± 6.87 vs. 6.83 ± 5.92 mL/h/m^2^; *p* = 0.005) (Figure 1A). In our limited number of measurements, neither skin nor body temperature correlated with measured TEWL values (Supplemental Figure S1). When we compared the two methods of estimating skin water loss, mean TEWL-based values were significantly lower than formula-based estimates in both shock groups (Figure 1B). In patients with cardiogenic shock, the mean 24 h skin water loss calculated using TEWL was 328.0 ± 214.5 mL, compared to 494.2 ± 111.2 mL with the formula-based method (*p* = 0.005). A similar pattern was observed in patients with septic shock, where TEWL-based skin water loss was 383.6 ± 271.1 mL/24 h, significantly lower than the formula-based estimate of 529.9 ± 196 mL/24 h (*p* = 0.009).

Moreover, direct comparison of the two methods revealed no statistically significant correlation between TEWL-based and formula-based estimates of skin water loss (R^2^ = 0.13, *p* = 0.07) (Figure 1C). To assess how the observed deviation between TEWL-based and formula-based values varies across different levels of skin water loss, we compared TEWL-based measurements with the relative deviation of formula-based estimates from TEWL-based measurements (Figure 1D). As previously noted, the mean of TEWL-based measurements was generally lower, with the majority of formula-based estimates exceeding TEWL values by 25% or more. Notably, we observed a significant positive correlation between TEWL values and the magnitude of deviation, indicating that at low levels of TEWL-assessed skin water loss, the formula tends to overestimate values by up to 50%. In contrast, at higher levels of transcutaneous water loss, the formula substantially underestimates actual loss-by almost 100%.

**Figure 2:**
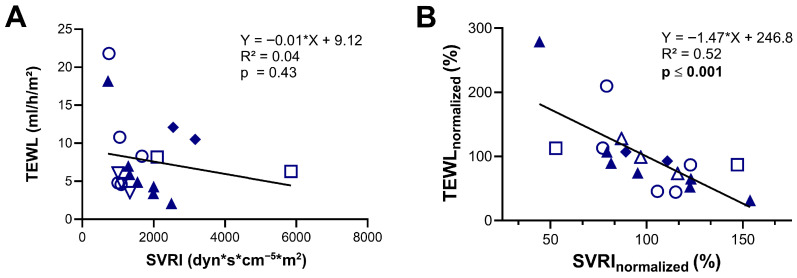
Assessment of transepidermal water loss (TEWL) as a predictor of systemic vascular resistance index (SVRI). (**A**) Correlation between TEWL values and SVRI measured using the PiCCO device. (**B**) Correlation between TEWL and SVRI values expressed as percentages of each patient’s individual mean (normalized data). Identical symbols represent repeated measurements in the same patient; color-filled symbols denote patients with cardiogenic shock. The dataset includes 9 individual measurements from 2 patients with cardiogenic shock and 10 from 4 patients with septic shock. Statistical analysis was performed using simple linear regression (**A**,**B**).

Furthermore, we evaluated if TEWL can be used as a surrogate marker for vascular tone by comparing TEWL measurements with simultaneous SVRI data obtained via the PiCCO^®^ device (Pulsion Medical Systems, Munich, Germany). Analysis of the whole cohort showed no significant correlation between the absolute values of these two simultaneously measured parameters (R^2^ = 0.04; *p* = 0.43) (Figure 2A). However, when normalized to each individual’s mean, a moderate yet statistically significant negative correlation was observed (R^2^ = 0.52, *p* ≤ 0.001), indicating that within individuals, lower vascular tone (low SVRI) was associated with higher TEWL values, and vice versa (Figure 2B).

## 4. Discussion

Our data indicate that the measurement of TEWL might broaden the assessment of ICU patients with septic or cardiogenic shock. Our findings suggest that TEWL measurement can be implemented in intensive care settings and may complement existing diagnostics in two dimensions: First, we found that TEWL provides an extremely simple tool to enhance the so far sparsely performed assessment of skin water loss for improving the quantification of the daily fluid balance. Additionally, on an intra-individual basis, the direct correlation between TEWL and vascular resistance indicates that directly measured skin water loss could be an additional tool for a non-invasive assessment of vascular tone.

While fluid balance charting may seem routine in ICU settings, it remains challenging. Mensink et al. provided compelling evidence that recorded fluid balances correlate poorly with actual changes in body weight [20]. These findings suggest that even automated fluid balance charting through electronic health records fails to provide accurate representations of actual fluid balances. The challenge is not only to accurately track all administered fluids but also in accounting for fluid losses beyond measurable outputs like urine and drainage fluids. Insensible fluid losses occurring through cutaneous evaporation and the respiratory tract are typically neither directly quantifiable nor routinely assessed due to methodological limitations. Therefore, several formulas are used in clinical practice to estimate these losses [10,21]. Cox et al. suggested that insensible water loss is approximately 10 mL/kg/day, serving as a simple and reasonably accurate rule of thumb for estimating basal insensible water loss in adults, with a 40% reduction if the patient is mechanically ventilated with active humidification [9]. When compared to TEWL-based measurements, this formula generally overestimated insensible water loss in our study. Furthermore, we observed no correlation between body or skin temperature and skin water loss as assessed by TEWL, challenging the validity of temperature-based correction factors used in various estimation formulas, including the one we applied. We see a clear need for additional research utilizing TEWL assessments in critically ill patients to validate and further explore these findings.

Altemeier et al. identified cool, diaphoretic skin as a poor prognostic indicator in patients with sepsis, published almost 70 years ago [22]. But despite its accessibility, the diagnostic potential of the skin remains underutilized in routine ICU practice. Even basic skin inspection without a technical device has been shown to provide prognostic information in patients with septic [23] and cardiogenic [24] shock: the mottling score is a bedside tool shown to be a strong predictor of mortality in septic patients [25]. The score is assessed by visually grading skin discolouration—typically over the knees—on a scale from 0 (no mottling) to 5 (severe mottling extending beyond the groin) [23]. Despite its prognostic significance, the mottling score is most referenced in critical care settings with limited resources [26,27]. Reflecting this, also the 2021 Surviving Sepsis Campaign guidelines recommend skin mottling as a surrogate marker for organ and tissue perfusion “when advanced hemodynamic monitoring is unavailable” [3]. One potential limitation of the mottling score is its subjective nature. Objective assessment of skin water loss with a numeric value may help address this drawback.

The simple measurement of TEWL with a handheld device, as in our study, may enhance bedside diagnostics and provide insights into skin microcirculatory function and vascular resistance. Yet, our data indicate that absolute TEWL values, due to high intraindividual variability, do not correlate significantly with SVRI. Only intraindividually normalized TEWL values demonstrated a statistically significant correlation with systemic vascular resistance derived from PiCCO^®^ measurements. However, the moderate R^2^ indicates that this association is weak. These findings therefore suggest that meaningful— interpretation is limited to longitudinal, within-patient assessments, and that TEWL should only be regarded as a weak surrogate marker of vascular resistance. Given the fast and non-invasive nature of TEWL measurement, this approach may nevertheless offer a safe and practical tool for additional patient monitoring. The skin contains a vascular bed whose diagnostic relevance in shock remains a subject of ongoing debate [28]. To date, it stands far behind sublingual microvessels, which have been assessed using automated microscopy in various studies [29,30], though these techniques still remain under debate and have not found widespread clinical adoption [31,32,33].

Our study has several notable limitations. This feasibility study includes measurements and calculations performed on only eight patients, which limits the generalizability of the findings and classifies the results as hypothesis-generating. Measurements of transepidermal water loss were conducted at single time points rather than continuously, preventing assessment of intraindividual changes over time. We want to emphasize that the device used in this study is not designed for continuous monitoring. The application of closely timed repeated measurements could mitigate this limitation; however, this approach is inherently resource-intensive and might be impractical for large-scale implementation. Moreover, all data were collected from a single intensive care unit without any randomization or standardization regarding the timing of measurements throughout the disease course. Importantly, we did not measure concurrent changes in total body weight as such, nor did we perform bioimpedance-based assessments of volume status. Therefore, we cannot infer a final statement if a TEWL-based approach surpasses the formula-based estimation of fluid losses. Future studies with larger, more diverse cohorts and controlled measurement protocols are required to validate these preliminary findings and further elucidate the underlying physiological principles in humans.

## 5. Conclusions

This feasibility study shows that transepidermal water loss (TEWL) can be reliably measured in critically ill patients with septic or cardiogenic shock and may offer additional value for assessing fluid balance and vascular tone. While absolute TEWL values did not correlate with systemic vascular resistance, intraindividual changes did—suggesting TEWL might serve as a dynamic, non-invasive monitoring tool. Although limited by sample size and single-center design, these findings are hypothesis-generating and support further research. TEWL measurement could complement current fluid management strategies and improve individualized care in the ICU.

## Figures and Tables

**Table 1:** Baseline characteristics of all 8 included patients.

Category	Parameter	
Patient Characteristics	Age (years ± SD)	64.3 ± 9.3
	Female sex	12.5%
	Body mass index (kg/m^2^ ± SD)	26.24 ± 5.11
	Cardiogenic shock	37.5%
	Septic shock	62.5%
Comorbidities (%)	Hypertension	50%
	Diabetes mellitus	25%
	Chronic kidney disease	62.5%
	Chronic heart failure	50%
	Chronic obstructive pulmonary disease	25%
	Stroke	12.5%
	Myocardial infarction	25%
	Active skin disease	0%
	Chronic liver disease	12.5%
	Malignancy	37.5%
	Smoking	50%
	Dyslipoproteinemia	62.5%
Laboratory Parameters on ICU-admission	Leukocyte count (×10^9^/L ± SD)	14.84 ± 10.4
	Lymphocyte count (% ± SD)	7.39 ± 7.8
	Platelet count (×10^9^/L ± SD)	220.88 ± 141.8
	Hemoglobine (g/dL)	10.57 ± 2.27
	Serum creatinine (mg/dL ± SD)	1.83 ± 1.25
	Total bilirubin (mg/dL ± SD)	0.90 ± 0.61
	Aspartate aminotransferase (mU/mL ± SD)	194.63 ± 152.0
	Alanine aminotransferase (mU/mL ± SD)	93.13 ± 62.2
	Lactate (mmol/L ± SD)	3.46 ± 2.2

**Table 2:** Maximum or minimum recorded laboratory values, maximum severity scores and clinical outcomes for all included patients.

Category	Parameter	
Laboratory Values during ICU stay	Lowest platelet count (×10^9^/L ± SD)	116.00 ± 83.6
	Peak serum creatinine (mg/dL ± SD)	3.23 ± 2.5
	Highest total bilirubin (mg/dL ± SD)	1.99 ± 2.3
	Highest C-reactive protein (mg/L ± SD)	227.38 ± 60.9
	Highest creatine kinase (U/L ± SD)	1574.25 ± 1680.4
	Highest troponin level (pg/mL ± SD)	59,820.88 ± 142,115.2
	Highest lactate (mmol/L ± SD)	6.51 ± 4.2
	Maximum vasopressor dose (μg/min ± SD)	69.79 ± 72.0
Severity Scores	Maximum APACHE II score	27 ± 8
	Maximum SOFA score	12 ± 3
Clinical Course and Outcomes	Total duration of mechanical ventilation (h ± SD)	167.40 ± 160.2
	Total ICU length of stay (h ± SD)	245.65 ± 197.1
	Total hospital length of stay (days ± SD)	26.50 ± 15.8
	Transferred alive from ICU (%)	62.5%
	Discharged alive (%)	62.5%

**Table 3:** Twenty-four-hour fluid intake and excretion in all included patients, stratified by type of shock (septic vs. cardiogenic). Data represent 14 (septic shock) and 12 (cardiogenic shock) measurements from 5 and 3 individual patients, respectively. Statistical analysis was performed using Welch’s *t*-test or the Mann–Whitney U test, as appropriate.

		Septic Shock	Cardiogenic Shock	*p*-Value
24 h-Fluid Intake	Parenteral (mL + SD)	4581 ± 1337	3012 ± 715.1	0.001
	Enteral (mL + SD)	136.6 ± 268.4	353.8 ± 371.8	0.11
	Total (mL + SD)	4718 ± 1412	3365 ± 898.9	0.01
24 h-Fluid Excretion	Urine Volume (mL + SD)	840.7 ± 872.7	896.9 ± 1176	0.89
	Ultrafiltrate (mL + SD)	425.8 ± 584.7	1728 ± 1454	0.03
	Formula-based Skin water loss (mL + SD)	529.9 ± 196	494.2 ± 111.2	0.96
	TEWL-based Skin water loss (mL + SD)	383.6 ± 271.1	328 ± 214.5	0.61
	Total (using Formula-based Skin WL) (mL + SD)	1721 ± 652.8	2646 ± 1144	0.02
	Total (using TEWL-based Skin WL) (mL + SD)	1650 ± 678.6	2521 ± 1080	0.02
24 h-Fluid Balance	Total (using Formula-based Skin WL) (mL + SD)	2997 ± 1492	719.7 ± 1564	0.001
	Total (using TEWL-based Skin WL) (mL + SD)	3068 ± 1428	844.6 ± 1502	0.67

## Data Availability

All data are presented in the manuscript and its accompanying files.

## Supplementary Materials

The following supporting information can be downloaded at: https://www.mdpi.com/article/10.3390/biomedicines13112644/s1, Figure S1: Correlation of TEWL with skin (A) and body (B) temperature. Table S1: Individual values from all included patients.

## Abbreviations

The following abbreviations are used in this manuscript:

ANOVA	Analysis of variance
APACHE II	Acute Physiology and Chronic Health Evaluation II
BMI	Body mass index
DRKS	Deutsches Register Klinischer Studien (German Clinical Trials Register)
ICU	Intensive care unit
SD	Standard deviation
SEM	Standard error of the mean
SOFA	Sequential Organ Failure Assessment
SVRI	Systemic vascular resistance index
TEWL	Transepidermal (or transepithelial) water loss
