# Supplementary material for: Assessing Vascular Tone and Fluid Balance in Septic and Cardiogenic Shock: A Feasibility Study on Skin Water Loss as a Diagnostic Tool
Source: Biomedicines. 2025 Oct 28;13(11):2644. doi: 10.3390/biomedicines13112644 (PMC12650400; doi:10.3390/biomedicines13112644)
Supplement: Supplementary file 1 [file biomedicines-13-02644-s001.zip › biomedicines-3880979-supplementary.pdf]

Supplemental Figure S1. Correlation of TEWL with skin (A) and body (B) temperature. The dataset includes 26 individual measurements from 3 patients with cardiogenic shock and 5 patients with septic shock. Statistical analysis was performed using simple linear regression.

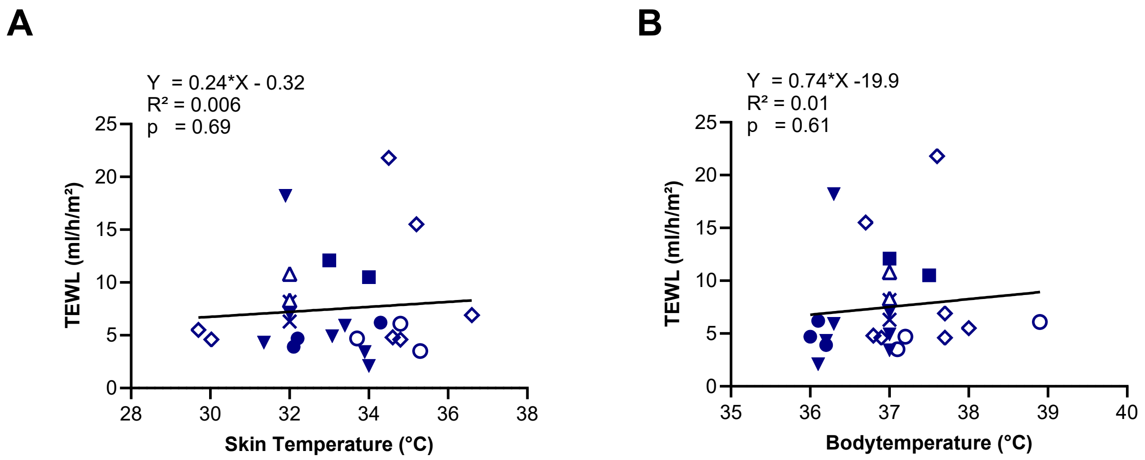

Supplemental Table S1

Individual values from all included patients

Measurement No.	Patient No.	Cardiogenic Shock (yes/no)	Septic Shock (yes/no)	Time Difference Between Measurements (h)	Body Surface Area (m^2)	RASS (Richmond Agitation-Sedation Scale)	APACHE II Score	SOFA Score	Leukocytes	Platelets	Hemoglobin	Creatinine	Urea	Bilirubin	ALAT	CRP	Sodium	Potassium	Calcium	Lactate	Glucose	Troponin	TEWL Forearm	TEWL Lower Limb	TEWL Trunk	TEWL Forehead	TEWL body total (ml/24h)	SVRI
1	1	y	n	0	2,2	-5	23	9	12.0	203	13,8	0,74	11	0,6	72	152	144	3,8	1,16	1,5	148	1998	5,26	4,76	3,32	9,4	248,2	-
2	1	y	n	23,1	2,2	-3	23	9	9,9	164	11,9	0,67	15	0,4	72	80	144	4,1	1,17	1,7	163	1484	3,08	3,37	4,31	5,9	203,3	-
3	1	y	n	48	2,2	-3	23	9	9,8	192	11,9	0,77	20	0,4	63	29	145	4,1	1,13	0,8	150	1189	8,9	5,57	5,7	5,5	324,9	-
4	2	n	y	0	1,8	-3	24	10	10,3	18	7,9	1,65	36	0,8	2638	83	147	4,4	1,08	1,1	188	229	26,4	17,5	8,6	14,6	668,0	-
5	2	n	y	24,2	1,8	-2	24	9	9,9	41	9	1,53	33	1,1	1560	103	147	4,2	1,1	1,1	127	156	4,9	5,7	4,5	10,2	236,4	-
6	2	n	y	48	1,8	-3	24	9	6,9	23	8,2	1,48	30	1,1	1002	148	145	4,5	1,1	0,8	124	111	4,8	6,9	3,9	24,2	299,4	-
7	2	n	y	71	1,8	-3	24	9	7,3	28	7,4	2	43	1,4	423	109	142	4,8	1,1	0,7	75	94	6,6	4,9	3	6,8	200,6	-
8	3	y	n	0	1,8	-5	20	15	7,8	99	13,3	1,3	16	2,3	47	27	145	3,6	1,21	1,1	97	156	3,6	1,6	1,8	2,1	89,0	2500
9	3	y	n	21,9	1,8	-5	20	17	10,8	93	13,6	2,41	23	5,7	47	167	149	4,8	1,13	1,4	151	56,8	36,2	7,9	19,3	21,4	787,7	720
10	3	y	n	47	1,8	-5	20	18	12,2	55	12,8	2,25	25	7	67	194	141	5,1	1,2	1,1	189	34	1,95	8,42	2,5	6,5	210,3	1550
11	3	y	n	73	1,8	-4	20	16	9,7	80	7,6	2,9	67	6,5	79	49	138	4,3	1,1		162	38	6,93	6,02	7,8	8,62	302,3	1290
12	3	y	n	96	1,8	-3	20	16	9,8	72	7,9	3,01	70	7,4	70	33	140	4,1	1,05	0,8	140	35	4,75	6,8	5	8,4	253,1	1326
13	3	y	n	118	1,8	-3	20	16	8,3	70	8,1	3,1	73	6,9	77	30	138	4,2	1,1	1	139	712	4,3	4,2	1,8	5,6	148,5	1992
14	3	y	n	144	1,8	-3	20	16	6,45	64	8,5	2,75	57	6,5	80	27	138	4,1	1,1	0,9	141	339	5	6,1	2,1	4,7	184,7	2000
15	4	n	y	0	2,2	-5	28	14	9,09	119	10	2,1	34	0,9	61	216	144	4,9	1,31	1,1	154	81	31,4	9,4	28,8	27	1152,8	750
16	4	n	y	21	2,2	-4	26	14	7,82	95	9,2	2,36	42	0,8	59	185	145	3,9	1,25	0,9	163	31	2,54	4,2	6,4	5,4	251,3	1000
17	4	n	y	45	2,2	-5	27	13	5,38	95	8,7	1,79	45	0,5	53	103	152	4,6	1,24	0,7	148	34	2,7	5,1	3,6	11,2	244,3	1090
18	5	n	y	0	1,45	-4	16	8	6,14	191	8,5	0,56	10	0,3	55	192	151	4,5	1,2	0,7	112	57	6,2	5,9	2,78	5,4	164,5	1115
19	5	n	y	19	1,45	-4	16	8	6,07	221	9,5	0,56	14	0,3	53	182	148	4,5	1,19	0,7	80	70	3,8	9,6	5,04	1,7	212,5	1000
20	5	n	y	43	1,45	-2	16	8	4,2	202	8,8	0,5	19	0,3	50	174	149	4,1	1,15	0,9	110	56	3,5	4,1	2,7	4,7	121,8	1336
21	6	y	n	0	2,18	0	21	6	23,3	519	13,1	0,7	10	0,5	66	250	140	4,7	1,19	2,3	235	79743	10,2	9,8	10,5	15,2	550,0	3165
22	6	y	n	26	2,18	-5	32	10	23,2	232	9,5	2,5	33	0,6	1173	91	145	4,7	1,16	1,7	180	74374	13,6	10,1	11,6	20,7	634,3	2549
23	7	n	y	0	2,12	-2	28	14	0	29	9,5	1,7	43	0,7	27	338	145	5,5	1,25	5,4	107	1585	10,5	11,2	10,6	11,7	549,0	1052
24	7	n	y	23	2,12	-5	33	16	0	69	9,7	1,5	36	0,8	41	349	146	4,9	1,09	1,2	111	1689	8,4	9,5	7,7	6,5	421,7	1672
25	8	n	y	0	2,44	-5	40	14	12,5	216	8,5	2,9	20	1,7	1544	191	151	5,7	1,14	7,4	380	6800	9,2	5,9	7,9	17,2	478,6	2100
26	8	n	y	21	2,44	-5	43	14	12,3	197	8,4	2,7	22	2	5007	174	153	6,3	1,05	9,4	140	32193	6,2	4,2	6,4	15,2	368,9	5853
